# Prevalence of Haemoplasma Infections in Stray Cats in Northern Italy

**DOI:** 10.1155/2014/298352

**Published:** 2014-02-23

**Authors:** Eva Spada, Daniela Proverbio, Paola Galluzzo, Alessandra Della Pepa, Giada Bagnagatti De Giorgi, Roberta Perego, Elisabetta Ferro

**Affiliations:** ^1^Reparto di Medicina Emotrasfusionale Veterinaria (REV), Dipartimento di Scienze Veterinarie per la Salute, la Produzione Animale e la Sicurezza Alimentare (VE.S.P.A.), Università degli Studi di Milano, Via G. Celoria, 10-20133 Milano, Italy; ^2^Centro di Referenza Nazionale per Anaplasma, Babesia, Rickettsia e Theileria (C.R.A.Ba.R.T), Istituto Zooprofilattico Sperimentale della Sicilia, Via G. Marinuzzi, 3-90129 Palermo, Italy

## Abstract

This study investigated the prevalence of feline haemoplasma infections in a number of stray cat colonies in Milan, Northern Italy. Blood samples from 260 stray cats were evaluated, with conventional PCR, for the presence of DNA associated with *Mycoplasma haemofelis* (Mhf) and “*Candidatus Mycoplasma haemominutum*” (CMhm). Odd ratios (OR) were calculated to identify risk factors for haemoplasma infections. PCR was positive in 86 out of 260 subjects (33.1%), with a prevalence of 10.8% (28/260 cats) for Mhf and 22.3% (58/260 cats) for CMhm. No coinfections were registered. There were significant associations between infections and season of sampling, that is, a negative association between winter sampling and a haemoplasma positive status (OR = 0.29, *P* = 0.001), or CMhm positive status (OR = 0.29, *P* = 0.01). Haemoplasma infections are common in stray cats in Milan. Thus, domestic cats with outdoor access should be routinely monitored and treated for ectoparasites to minimize risks of disease acquisition. Moreover, as these infections are transmitted via blood, feline blood donors from this area should be screened by PCR and preferably be drawn from a population of indoor cats regularly treated for fleas.

## 1. Introduction

Haemotropic mycoplasmas (haemoplasmas) are bacterial organisms without cell walls that attach to and grow on the surface of red blood cells. Three feline haemoplasma species are described: *Mycoplasma haemofelis* (Mhf), “*Candidatus Mycoplasma haemominutum*” (CMhm), and “*Candidatus Mycoplasma turicensis*” (CMt) [1]. These feline haemoplasmas are the causative agents of infectious anemia in cats and in several mammalian species. There is also potential interspecies transmission of some of these agents as recorded from cats to immunocompromised dogs [2]. Their zoonotic potential has recently been substantiated by the molecular identification of a feline haemoplasma isolate (Mhf) in an HIV-positive immunocompromised human patient [3]. The three feline haemoplasma species have different pathogenicities, Mhf often resulting in haemolysis and severe anaemia in contrast to CMhm and CMt which are less pathogenic [4].

Although several studies worldwide have reported on the epidemiology of feline hemoplasmosis in sick or healthy client-owned pet cats with prevalences ranging from 7.2% to 45.8% [5–19] and few studies have focused on stray cat (with prevalences from 11.5% to 60% [20–24]), there have been no studies investigating stray cats in Northern Italy. Information on regional prevalence of haemoplasmas could be used to limit the spread of diseases in feline populations and for predicting the likelihood of infection in cats presented with anemia.

The transmission of haemoplasmas is still poorly understood. The vector potential of *Ctenocephalides felis* has been demonstrated in experimental Mhf infections [25], and stray cats may play a role in multiplying the organism in fleas that then infest pet cats and dogs and human beings. Direct transmission, by aggressive interaction of cats, or interspecies transmission might play a role in the epidemiology of these organisms. In addition, haemoplasmas can be directly transmitted through intravenous infusion of infected blood [4, 26] and have been shown to survive for up to 1 week in stored blood products [27]. As administration of fresh or stored whole blood becoming more common in feline medicine, the knowledge of the regional prevalence of blood transmitted pathogens in owned and stray cats that share the same environment and parasites is increasingly important. This would provide useful information for evaluating the risks of transmission of blood-borne infections from potential blood donors and in the development of optimal screening protocols in blood donors.

The aim of this study was to evaluate, using a conventional PCR assay, the prevalence of Mhf and CMhm infections in stray cats from colonies in Milan and to identify possible risk factors for these infections.

## 2. Materials and Methods

### 2.1. Sample Population and Data Collection

During a 2-year collection period (January 2008 to January 2010), blood samples were taken from 260 stray cats from urban colonies in Milan (Northern Italy), under a trap-neuter-release (TNR) program approved by the local authority of the city council. The program was conducted as previously described [28, 29].

Age (estimated based on dentition, animals <6 months of age were considered juvenile, whereas all others were considered adult), gender (male or female), origin (provenance area of colonies), and body condition score (BCS 4–6, indicating normal weight, 1–3 underweight) were recorded together with data obtained from physical examination of the cats (healthy or unhealthy). Unhealthy cats were defined as cats with one or more of the following clinical abnormalities: lymph node enlargement, pale mucous membranes, stomatitis, or signs of ocular and respiratory infections.

The seasonal analysis based on the date of sample collection was grouped as follows: winter (January, February, and March), spring (April, May, and June), summer (July, August, and September), autumn (October, November, and December). The cats were not systematically examined for the presence of ticks or fleas and so rates of ectoparasitism were not recorded.

### 2.2. Haematological and Serological Analyses

Blood samples were collected aseptically from the jugular vein while cats were anaesthetized for neutering and placed in EDTA-treated tubes and in serum separator tubes.

Within 24 h of sample collection, a complete blood count (CBC) was performed on whole blood using an ADVIA 120 System (Siemens Healthcare Diagnostics, Milan, Italy). Cats were categorized in terms of presence or absence of anaemia (HCT < 24%), leukopenia (WBCs count < 10,570/*μ*L), leukocytosis (WBC > 14,390/*μ*L), and thrombocytopenia (PLT < 200,670/*μ*L) [30]. Surplus blood was stored at −20°C to use later in PCR assay.

Following separation, serum samples were tested for antibodies to FIV (relative to gp40 and p24 FIV antigens) and for FeLV p27 antigen with a commercial enzyme-linked immunosorbent assay (ELISA) kit (Snap FeLV/FIV Combo Plus Test; Idexx Laboratories, Hoofddorp, The Netherlands). *Toxoplasma gondii* sera IgG antibodies were detected using indirect fluorescent antibody tests (IFAT) performed with a commercial kit (Fuller-laboratories, Fullerton, CA, USA). Titres ≥1 : 64 were considered seroreactive and, therefore, indicative of *T. gondii* exposure. The results of these serological tests have been already published [28] and were reanalyzed with the present results.

### 2.3. PCR Assay

Conventional PCR was performed on blood samples to amplify DNA associated with haemoplasmas (Mhf and CMhm). The reaction mixture included 2 *μ*L of template DNA, 0.25 mM dNTPs, 0.4 mM of each primer, 1x reaction buffer, and 2.5 U *Tap* DNA polymerase (GoTaq DNA Polymerase, Promega, Madison, WI, USA). The volume of this mixture was adjusted to 25 *μ*L with sterile water. Primers were used that target part of the 16S rRNA gene, producing a 170 base pair (bp) product from Mhf and 193 bp amplicon from MChm (forward primer, 5′-ACG AAA GTC TGA TGG AGC AAT A-3′ and reverse primer 5′-ACG CCC AAT AAA TCC GRA TAA T-3′) [6, 31]. PCR reactions were performed using an automated thermocycler. PCR products were resolved using 2% agarose gels and fragment size was estimated using a DNA molecular weight marker (50 bp DNA Ladder; Promega Madison, WI, USA). Control reactions were done in the absence of template DNA to rule out contaminations during PCR.

### 2.4. Statistical Analysis

Association between the three groups of haemoplasma status (haemoplasma positive, Mhf positive only, and CMhm positive only) and categorical variables (age, gender, colony of origin, BCS, season of sampling, health status, presence or absence of selected clinical and CBC abnormalities, FeLV/FIV status, and *T. gondii* test results) were analysed by univariate analysis using the chi-squared test (cell frequencies of >5) or Fisher's exact test (cell frequencies of ≤5). Any parameters statistically linked to positive PCR results were used in a logistic regression model to test for independent risk factors associated with infection.

Descriptive statistics (including minimum, maximum, mean, median, and standard deviation (SD)) were obtained for the continuous variables RBCs count, HCT, HB, WBC count, and PLT count values. Distribution of the data for normality was assessed with Kolmogorov Smirnov test, and a *t*-test or a Mann-Whitney *U* test, respectively, was used to test the differences between the feline haemoplasma positive and negative cats depending on whether data was normally distributed or not.

Associations were considered statistically significant when *P* < 0.05. Both the *P* value and odds ratio (OR) with 95% confidence interval (CI) are reported. Data were analyzed using MedCalc Software (version 12.3.0; Mariakerke, Belgium).

## 3. Results

Haemoplasmic DNA was detected in 33.1% (86/260, 95% CI 26.5–40.9%) of blood samples. Of the positive samples, 28 cats (10.8%, 95% CI 7.2–15.6%) were infected with Mhf and 58 cats (22.3%, 95% CI 16.9–28.8%) were infected with CMhm. No comorbid infections were registered.

Characteristics of the population and association between risk factors and haemoplasma PCR status (negative or positive) are reported in Table 1 and between Mhf alone and CMhm alone positive results in Table 2.

None of the risk factors were associated with the PCR results for haemoplasmas (Tables 1 and 2), with the exception of negative associations at univariate and multivariate analysis between winter season of sampling and haemoplasma positive status (*P* = 0.01, OR = 0.29, 95% CI = 0.14–0.61, *P* = 0.001) and CMhm positive status (*P* = 0.01, OR = 0.29, 95% CI = 0.12–0.70, *P* = 0.01).

**Figure 1:**
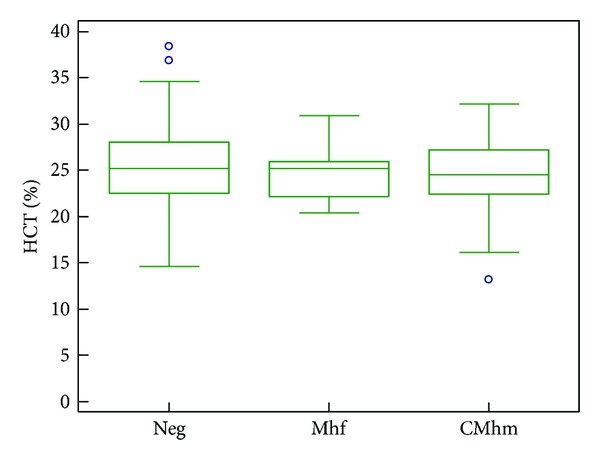
HCT values of Italian stray cats grouped by haemoplasma infectious status. Boxes represent the 25th, 50th (median), and 75th quartiles with whiskers extending to the greatest and smallest values. Blue circles indicate outliers (cases with values greater than 1.5 box lengths from the upper or lower edge of the box). CMhm: “*Candidatus M. haemominutum*” positive; Mhf: *M. haemofelis* positive. The HCT values of CMhm group and the Mhf group were not significantly lower than the negative group. The HCT values of the Mhf group were also not significantly lower than the CMhm group.

CBC results from 150/260 cats are reported in Table 3. Data was normally distributed according to Kolmogorov Smirnov test. No significant associations were found with CBC abnormalities and haemoplasma PCR-positive results (Figure 1) or in the number of anaemic and nonanaemic cats using a *t*-test.

## 4. Discussion

We present the first study investigating the prevalence of haemoplasmas in urban stray colony cats from the city of Milan in Northern Italy.

The prevalence of haemoplasma infection in our sample showed a similar pattern to that reported worldwide in client-owned [5–7, 9–15, 17–19] and stray cats [19–23]. CMhm infection is reported to be the most common infection, ranging from 8% in Arizona [21] to 41.6% in Portugal [19]; Mhf infection was less common ranging from 0.5% in Switzerland [9] to 12.8% in Portugal [19] and dual infection was absent or rare (no more than 5.4% as found in Korea) [22].

The prevalence of haemoplasmas in stray cats in our study (33.1%) was higher than that reported in other studies on stray cats performed worldwide (Table 4). Differences in prevalence among countries can be explained by geographical variation, such as climate, vector distribution, and the cat population surveyed. Moreover, direct comparisons of prevalence results are of limited value because of the characteristics of sample of cat populations investigated (number of cats included in the study, healthy versus sick cats), inclusion criteria, diagnostic techniques (molecular tools versus microscopical detection), different statistical methods, stage of infection (acute versus chronic), or a combination of all them, resulting in differences between studies.

Stray cats in this study had higher rates of infections than those reported for pet cats in Northern Italy [12], in which 18.9% of PCR tested cats were reported to be haemoplasma positive. A high incidence of haemoplasmas in stray cats is not surprising as outdoor access is a recognized risk factor for infection. For example, in a study on haemoplasma in client-owned cats from Barcelona (Spain), outdoor access was found to be a risk factor for infection (OR = 3.8) [15]. Additionally, in a study involving feline blood donors from the USA, the prevalence of haemoplasmas was 19.7% in domestic cats with outdoor access and only 3.6% in domestic cats not allowed outdoors [8]. Free-ranging cats may have more exposure to bloodsucking arthropods and exhibit a higher fighting activity than owned indoor cats; thus they might experience a higher infection risk for haemoplasmas.

In our study there was a negative statistical association between sampling in the winter season and haemoplasma PCR-positive status and CMhm positive status. This may be due to reduced outdoor activity of fleas in winter seasons, although this hypothesis is purely speculative. There were no statistically significant differences or any relationship between the presence of haemoplasma infection and anaemia status or HCT levels between positive and negative haemoplasma group. This finding was somewhat surprising and was in agreement with studies undertaken in client-owned cats in Switzerland [9] and in Italy [12], which also found no association between haemoplasma infection and anaemia or HCT variations. In addition, other studies have failed to demonstrate a significant difference in prevalence rates of haemoplasma between healthy and anaemic cats [10, 32]. These results could be explained by the higher prevalence of CMhm (a less pathogenic species than Mhf) in this and previous studies or by the fact that the stage of infection is not known in our cats with a positive PCR result, since chronically infected cats that recover from acute illness may be asymptomatic. Another explanation for the lack of association between infection status and the presence of anaemia in the present study could be that the HCT was known only for 150 of the 260 cats tested.

Epidemiological data on haemoplasma are particularly important in areas where feline blood donor programmes are active, as in Milan where a donor programme has been running since 2010 at University Veterinary Transfusion Unit (REV, Reparto di Medicina Emotrasfusionale Veterinaria). Feline haemoplasmas can be directly transmitted by intravenous infusion of fresh EDTA-anticoagulated blood [26], heparinized blood [4], and by infusion of blood stored in CPDA-1 solution for 1 h (Mhf) and 1 week (CMhm) [27]. Cats do not reliably eliminate the organism following infection [26] and most infections with CMhm are chronic and asymptomatic [1]. A significant number of asymptomatic cats are positive for haemoplasma infection [16] and may play a role, along with infected cats, in the maintenance of haemoplasma infection within a population. All these characteristics of feline haemotropic mycoplasma infection need to be considered when choosing potential blood donors.

The limitations of this study include the lack of information on “*Candidatus Mycoplasma turicensis*” in our study population. Risk factors analyzed were not available for all 260 cats. Lastly, statistical analysis was limited in some groups because of the sample size and so some conclusions or associations may be affected by type I errors; that is, no difference between haemoplasma positive and negative groups was observed due to insufficient sample size; for example, no association was recorded between anaemia and a positive haemoplasma result because of the low number of Mhf positive cats (28/260). Regardless of these limitations, we believe that this study provides new and useful information on feline haemoplasma infections in stray cats in Italy.

## 5. Conclusion

From this study it can be concluded that feline haemotropic mycoplasma Mhf and CMhm are common in the stray cat population of Milan. Indeed, pet cats with outdoor access in this region should be regularly monitored and treated for ectoparasites to minimize health risks. Importantly, feline blood donors in this area should undergo PCR screening for these infections before donations and preferably donors should be drawn from exclusively indoor cats that receive regular flea control.

## Figures and Tables

**Table 1:** Sample characteristics of 260 stray cats testing positive and negative for haemoplasma infection by conventional PCR in Northern Italy.

Factor	Category	Total number	PCR		*P* value
		(%)	Negative (%)	Positive (%)	
Origin of the cats (*n* = 260)	Zone 1	3 (1.2%)	3/174 (1.7%)	0/86 (0.0%)	*P* = 0.54
	Zone 2	11 (4.2%)	6/174 (3.4%)	5/86 (5.8%)	*P* = 0.57
	Zone 4	108 (41.5%)	68/174 (39.1%)	40/86 (46.5%)	*P* = 0.31
	Zone 5	12 (4.6%)	8/174 (4.6%)	4/86 (4.7%)	*P* = 0.77
	Zone 6	27 (10.4%)	15/174 (8.6%)	12/86 (14.0%)	*P* = 0.27
	Zone 7	55 (21.2%)	41/174 (23/6%)	14/86 (16.3%)	*P* = 0.23
	Zone 8	22 (8.5%)	14/174 (8.0%)	8/86 (9.3%)	*P* = 0.92
	Zone 9	22 (8.5%)	19/174 (10.9%)	3/86 (3.5%)	*P* = 0.07

Age (*n* = 260)	Juvenile (≤6 months)	118 (45.4%)	81/174 (46.6%)	37/86 (43.0%)	*P* = 0.69
	Adult (>6 months)	142 (54.6%)	93/174 (53.4%)	49/86 (57.0%)	

Gender (*n* = 260)	Male	90 (34.6%)	30/174 (17.2%)	60/86 (69.8%)	*P* = 0.37
	Female	170 (65.4%)	144/174 (82.7%)	26/86 (30.2%)	

BCS (*n* = 243)	Poor (1–3/9)	18 (7.4%)	10/165 (6.1%)	8/78 (10.3%)	*P* = 0.37
	Good (4–6/9)	225 (92.6%)	155/165 (93.9%)	70/78 (89.7%)	*P* = 0.54

Seasons (*n* = 260)	Winter	64 (24.6%)	54/64 (94.4%)	10/64 (15.6%)	***P=0.01***
					OR = 0.29
					CI = 0.14–0.61
					P=0.001*
	Spring	69 (26.5%)	38/69 (55.1%)	31/69 (44.9%)	*P* = 0.09
	Summer	31 (11.9%)	23/31 (74.2%)	8/31 (25.8%)	*P* = 0.54
	Autumn	96 (36.9%)	59/96 (61.5%)	37/96 (38.5%)	*P* = 0.40

Health status (*n* = 260)	Healthy	72 (27.7%)	47/174 (27.0%)	25/86 (29.1%)	*P* = 0.84
	Unhealthy	188 (72.3%)	127/174 (73.0%)	61/86 (70.9%)	

Clinical abnormalities in unhealthy cats (*n* = 188)	Lymph node enlargement	133 (70.7%)	87/102 (85.3%)	46/86 (53.5%)	*P* = 0.69
	Pale mucous membranes	14 (7.4%)	11/102 (10.8%)	3/86 (3.5%)	*P* = 0.51
	Stomatitis	101 (53.7%)	71/102 (69.6%)	30/86 (34.9%)	*P* = 0.43
	Signs of respiratory tract infection	22 (11.7%)	12/102 (11.8%)	10/86 (11.6%)	*P* = 0.29
	Signs of ocular infection	40 (21.3%)	26/102 (25.5%)	14/86 (16.3%)	*P* = 0.92

CBC abnormalities (*n* = 150)	Anaemia	62 (41.3%)	33/91 (36.3%)	29/59 (49.2%)	*P* = 0.69
	Leukopenia	15 (10.0%)	11/91 (12.1%)	4/59 (6.8%)	*P* = 0.56
	Leukocytosis	5 (3.3%)	3/91 (3.3%)	2/59 (3.4%)	*P* = 0.66
	Thrombocytopenia	10 (6.7%)	4/91 (4.4%)	6/59 (10.2%)	*P* = 0.30

FIV test results (*n* = 166)	Positive	13 (7.8%)	8/102 (7.8%)	5/64 (7.8%)	*P* = 0.82
	Negative	153 (92.2%)	94/102 (92.2%)	59/64 (92.2%)	

FeLV test results (*n* = 166)	Positive	6 (3.6%)	4/102 (3.9%)	2/64 (3.1%)	*P* = 0.90
	Negative	160 (96.4%)	98/102 (96.1%)	62/64 (96.9%)	

*T. gondii* test results (*n* = 113)	Positive	31 (27.4%)	18/87 (20.7%)	13/46 (28.3%)	*P* = 0.92
	Negative	82 (27.4%)	49/87 (56.3%)	33/46 (71.7%)	

PCR: polymerase chain reaction, BCS: body condition score, CBC: complete blood count, CI: 95% confidence interval, OR: odds ratio, FeLV: feline leukemia virus, and FIV: feline immunodeficiency virus.

*P* values in bold are statistically significant (*P* < 0.05).

*Results from multivariate logistic regression analysis.

**Table 2:** Characteristics of 28 Mhf PCR positive stray cats and 58 CMhm PCR positive stray cats in Northern Italy.

Factor	Category		PCR positive results		
		Mhf *n* (%)	*P* value	CMhm *n* (%)	*P* value
Origin of the cats	Zone 1	0/3 (0.0%)	*P* = 0.63	0/3 (0.0%)	*P* = 0.47
	Zone 2	1/11 (9.1%)		4/11 (36.4%)	
	Zone 4	12/108 (11.1%)		28/108 (25.9%)	
	Zone 5	1/12 (8.3%)		3/12 (25.0%)	
	Zone 6	6/27 (22.2%)		6/27 (22.2%)	
	Zone 7	5/55 (9.1%)		9/55 (16.4%)	
	Zone 8	2/22 (9.1%)		6/22 (27.3%)	
	Zone 9	1/22 (4.5%)		2/22 (9.1%)	

Age	Juvenile (≤6 months)	13/118 (11.0%)	*P* = 0.93	24/118 (20.3%)	*P* = 0.59
	Adult (>6 months)	15/142 (10.6%)		34/142 (23.9%)	

Gender	Male	11/90 (12.2%)	*P* = 0.15	15/90 (16.7%)	*P* = 0.15
	Female	17/170 (10.0%)		43/170 (25.3%)	

BCS	Poor (1–3/9)	3/18 (16.7%)	*P* = 0.46	5/18 (27.8%)	*P* = 0.84
	Good (4–6/9)	191/225 (84.9%)		51/225 (22.7%)	

Seasons	Winter	4/64 (6.3%)	*P* = 0.27	6/64 (9.4%)	***P=0.01***
					OR = 0.29*
					CI = 0.12–0.70*
					P=0.01*
	Spring	10/69 (14.5%)	*P* = 0.35	21/69 (30.4%)	*P* = 0.08
	Summer	2/31 (6.5%)	*P* = 0.60	6/31 (19.4%)	*P* = 0.85
	Autumn	12/96 (12.5%)	*P* = 0.63	25/96 (26.0%)	*P* = 0.34

Health status	Healthy	9/72 (12.5%)	*P* = 0.74	16/72 (22.2%)	*P* = 0.88
	Unhealthy	19/188 (10.1%)		42/188 (22.3%)	

Clinical abnormalities in unhealthy cats	Lymph node enlargement	13/133 (9.8%)	*P* = 0.74	33/133 (24.8%)	*P* = 0.40
	Pale mucous membranes	1/14 (7.1%)	*P* = 0.99	2/14 (14.3%)	*P* = 0.68
	Stomatitis	11/101 (10.9%)	*P* = 0.88	19/101 (18.8%)	*P* = 0.35
	Signs of respiratory tract infection	3/22 (14.1%)	*P* = 0.93	7/22 (31.8%)	*P* = 0.39
	Signs of ocular infection	3/40 (7.5%)	*P* = 0.65	11/40 (27.5%)	*P* = 0.52

CBC abnormalities	Anaemia	7/69 (10.1%)	*P* = 0.81	22/69 (31.9%)	*P* = 0.69
	Leukopenia	0/14 (0.0%)	*P* = 0.40	4/14 (28.6%)	*P* = 0.81
	Leukocytosis	0/5 (0.0%)	*P* = 1.00	2/5 (40.0%)	*P* = 0.97
	Thrombocytopenia	1/10 (10.0%)	*P* = 0.59	5/10 (50.0%)	*P* = 0.27

FIV test results	Positive	1/13 (7.7%)	*P* = 0.83	4/13 (30.8%)	*P* = 0.84
	Negative	15/150 (10.0%)		44/150 (29.3%)	

FeLV test results	Positive	1/6 (16.7%)	*P* = 0.90	1/6 (16.7%)	*P* = 0.81
	Negative	15/157 (9.6%)		47/157 (29.9%)	

*T. gondii* test results	Positive	6/31 (19.4%)	*P* = 0.14	7/31 (22.6%)	*P* = 0.38
	Negative	6/81 (7.4%)		27/81 (33.3%)	

PCR: polymerase chain reaction; BCS: body condition score; CBC: complete blood count; CI: 95% confidence interval; OR: odds ratio; FeLV: feline leukemia virus; FIV: feline immunodeficiency virus. *P* values in bold are statistically significant (*P* < 0.05).

*Results from multivariate logistic regression analysis.

**Table 3:** Selected haematological findings in subgroups of haemoplasma PCR-positive and PCR-negative cats.

Variable, reference range	Number of cats	Mean	SD	Median	Lowest value	Highest value
	PCR-positive for all haemoplasma					
WBC	59	11.019	4.427	10.720	1.558	23.240
RBC	59	6.406	1.059	6.300	2.100	8.750
HB	59	9.1	3.7	8.9	4.7	12.4
HCT	59	24.7	1.4	24.7	13.2	32.2
PLT	59	369	145	380	90	693

	PCR-negative for all haemoplasma					
WBC	91	10.815	3.957	10.440	1.516	22.870
RBC	91	6.555	1.113	6.550	3.780	9.190
HB	91	25.5	1.7	9.2	14.6	13.2
HCT	91	9.4	4.4	25.2	4.2	38.4
PLT	91	378	141	367	119	800

	PCR-positive Mhf					
WBC	15	10.547	3.062	9.990	6.400	15.460
RBC	15	6.541	800	6.310	5.610	8.460
HB	15	9.4	1.2	9.4	7.6	12.4
HCT	15	24.8	3.1	25.2	20.4	30.9
PLT	15	371	122	376	135	693

	PCR-positive CMhm					
WBC	43	11.181	4.825	10.790	1558	23.240
RBC	43	6.360	1.138	6.285	2.100	8.750
HB	43	9.0	1.5	8.9	4.7	12.1
HCT	43	24.6	4	24.5	13.2	32.2
PLT	43	369	154	386	90	662

WBC: white blood cell; RBC: red blood cells; HB: haemoglobin; HCT: haematocrit; PLT: platelet.

**Table 4:** Prevalence of haemoplasma infection in stray cats in worldwide studies.

Sample source (number of tested cats) [Ref]	Positive samples		
	Total prevalence	Mhf	CMhm
Italy current study (260)	33.1%	10.8%	22.3%
USA (Florida) (484) [20]	20.5%	8.3%	12.2%
Korea (331) [22]	14.5%	4.2%	10.3%
USA (Arizona) (112) [21]	27.7%	4.5%	8.0%
Ireland (75) [23]	17.3%	1.3%	13.3%
Canada (96) [24]	11.5%	3.1%	8.4%

Mhf: *Mycoplasma haemofelis*; CMhm: “*Candidatus Mycoplasma haemominutum*.”

## References

[1] Messick JB, Harvey JW, Greene CE (2012). Hemotropic mycoplasmosis (Hemobartonellosis). *Infectious Diseases of the Dog and Cat*.

[2] Obara H, Fujihara M, Watanabe Y, Ono HK, Harasawa R (2011). A feline hemoplasma, “*Candidatus* Mycoplasma haemominutum”, detected in dog in Japan. *Journal of Veterinary Medical Science*.

[3] dos Santos AP, dos Santos RP, Biondo AW (2008). Hemoplasma infection in HIV-positive patient, Brazil. *Emerging Infectious Diseases*.

[4] Tasker S, Peters IR, Papasouliotis K (2009). Description of outcomes of experimental infection with feline haemoplasmas: copy numbers, haematology, Coombs’ testing and blood glucose concentrations. *Veterinary Microbiology*.

[5] Tasker S, Braddock JA, Baral R (2004). Diagnosis of feline haemoplasma infection in Australian cats using a real-time PCR assay. *Journal of Feline Medicine and Surgery*.

[6] Kewish KE, Appleyard GD, Myers SL, Kidney BA, Jackson ML (2004). *Mycoplasma haemofelis* and *Mycoplasma haemominutum* detection by polymerase chain reaction in cats from Saskatchewan and Alberta. *Canadian Veterinary Journal*.

[7] Lappin MR, Griffin B, Brunt J (2006). Prevalence of *Bartonella* species, *Haemoplasma* species, *Ehrlichia* species, *Anaplasma phagocytophilum*, and *Neorickettsia risticii* DNA in the blood of cats and their fleas in the United States. *Journal of Feline Medicine and Surgery*.

[8] Hackett TB, Jensen WA, Lehman TL (2006). Prevalence of DNA of *Mycoplasma haemofelis*, “*Candidatus Mycoplasma haemominutum*”; *Ananplasma phagocytophilum*, and species of *Bartonella, Neorickettsia*, and *Ehrlichia* in cats used as blood donors in the United States. *Journal of the American Veterinary Medical Association*.

[9] Willi B, Boretti FS, Baumgartner C (2006). Prevalence, risk factor analysis, and follow-up of infections caused by three feline hemoplasma species in cats in Switzerland. *Journal of Clinical Microbiology*.

[10] Bauer N, Balzer H-J, Thüre S, Moritz A (2008). Prevalence of feline haemotropic mycoplasmas in convenience samples of cats in Germany. *Journal of Feline Medicine and Surgery*.

[11] Peters IR, Helps CR, Willi B, Hofmann-Lehmann R, Tasker S (2008). The prevalence of three species of feline haemoplasmas in samples submitted to a diagnostics service as determined by three novel real-time duplex PCR assays. *Veterinary Microbiology*.

[12] Gentilini F, Novacco M, Turba ME, Willi B, Bacci ML, Hofmann-Lehmann R (2009). Use of combined conventional and real-time PCR to determine the epidemiology of feline haemoplasma infections in northern Italy. *Journal of Feline Medicine and Surgery*.

[13] Tanahara M, Miyamoto S, Nishio T (2010). An epidemiological survey of feline hemoplasma infection in Japan. *Journal of Veterinary Medical Science*.

[14] Barrs VR, Beatty JA, Wilson BJ (2010). Prevalence of *Bartonella* species, *Rickettsia felis*, *Haemoplasmas* and the *Ehrlichia* group in the blood of cats and fleas in eastern Australia. *Australian Veterinary Journal*.

[15] Roura X, Peters IR, Altet L (2010). Prevalence of hemotropic mycoplasmas in healthy and unhealthy cats and dogs in Spain. *Journal of Veterinary Diagnostic Investigation*.

[16] Bennett AD, Gunn-Moore DA, Brewer M, Lappin MR (2011). Prevalence of *Bartonella* species, Haemoplasmas and *Toxoplasma gondii* in cats in Scotland. *Journal of Feline Medicine and Surgery*.

[17] Lobetti R, Lappin MR (2012). Prevalence of *Toxoplasma gondii, Bartonella* species and haemoplasma infection in cats in South Africa. *Journal of Feline Medicine and Surgery*.

[18] Assarasakorn S, Veir JK, Hawley JR (2012). Prevalence of *Bartonella* species, hemoplasmas, and *Rickettsia felis* DNA in blood and fleas of cats in Bangkok, Thailand. *Research in Veterinary Science*.

[19] Martínez-Díaz VL, Silvestre-Ferreira AC, Vilhena H (2013). Prevalence and co-infection of haemotropic mycoplasmas in Portuguese cats by real-time polymerase chain reaction. *Journal of Feline Medicine and Surgery*.

[20] Luria BJ, Levy JK, Lappin MR (2004). Prevalence of infectious diseases in feral cats in Northern Florida. *Journal of Feline Medicine and Surgery*.

[21] Eberhardt JM, Neal K, Shackelford T, Lappin MR (2006). Prevalence of selected infectious disease agents in cats from Arizona. *Journal of Feline Medicine and Surgery*.

[22] Yu D-H, Kim H-W, Desai AR (2007). Molecular detection of feline hemoplasmas in feral cats in Korea. *Journal of Veterinary Medical Science*.

[23] Juvet F, Lappin MR, Brennan S, Mooney CT (2010). Prevalence of selected infectious agents in cats in Ireland. *Journal of Feline Medicine and Surgery*.

[24] Stojanovic V, Foley P (2011). Infectious disease prevalence in a feral cat population on Prince Edward Island, Canada. *Canadian Veterinary Journal*.

[25] Woods JE, Brewer MM, Hawley JR, Wisnewski N, Lappin MR (2005). Evaluation of experimental transmission of *Candidatus Mycoplasma haemominutum* and *Mycoplasma haemofelis* by Ctenocephalides felis to cats. *The American Journal of Veterinary Research*.

[26] Westfall DS, Jensen WA, Reagan WJ, Radecki SV, Lappin MR (2001). Inoculation of two genotypes of *Hemobartonella felis* (California and Ohio variants) to induce infection in cats and the response to treatment with azithromycin. *The American Journal of Veterinary Research*.

[27] Gary AT, Richmond HL, Tasker S, Hackett TB, Lappin MR (2006). Survival of *Mycoplasma haemofelis* and “*Candidatus Mycoplasma haemominutum*” in blood of cats used for transfusions. *Journal of Feline Medicine and Surgery*.

[28] Spada E, Proverbio D, Della Pepa A (2012). Seroprevalence of feline immunodeficiency virus, feline leukaemia virus and Toxoplasma gondii in stray cat colonies in northern Italy and correlation with clinical and laboratory data. *Journal of Feline Medicine and Surgery*.

[29] Spada E, Proverbio D, Della Pepa A (2013). Prevalence of faecal-borne parasites in colony stray cats in northern Italy. *Journal of Feline Medicine and Surgery*.

[30] Moritz A, Fickenscher Y, Meyer K, Failing K, Weiss DJ (2004). Canine and feline hematology reference values for the ADVIA 120 hematology system. *Veterinary Clinical Pathology*.

[31] Jensen WA, Lappin MR, Kamkar S, Reagan WJ (2001). Use of a polymerase chain reaction assay to detect and differentiate two strains of *Haemobartonella felis* in naturally infected cats. *The American Journal of Veterinary Research*.

[32] Ishak AM, Radecki S, Lappin MR (2007). Prevalence of *Mycoplasma haemofelis*, “*Candidatus Mycoplasma haemominutum*”, *Bartonella* species, *Ehrlichia* species, and *Anaplasma phagocytophilum* DNA in the blood of cats with anemia. *Journal of Feline Medicine and Surgery*.

